# SIRPα Mismatch Is Associated With Relapse Protection and Chronic Graft-Versus-Host Disease After Related Hematopoietic Stem Cell Transplantation for Lymphoid Malignancies

**DOI:** 10.3389/fimmu.2022.904718

**Published:** 2022-07-07

**Authors:** Rima M. Saliba, Samer A. Srour, Uri Greenbaum, Qing Ma, Yudith Carmazzi, Michael Moller, Janet Wood, Stefan O. Ciurea, Piyanuch Kongtim, Gabriela Rondon, Dan Li, Supawee Saengboon, Amin M. Alousi, Katayoun Rezvani, Elizabeth J. Shpall, Kai Cao, Richard E. Champlin, Jun Zou

**Affiliations:** ^1^ Department of Stem Cell Transplantation and Cellular Therapy, The University of Texas MD Anderson Cancer Center, Houston, TX, United States; ^2^ Department of Hematology, Soroka University Medical Center, Beer Sheva, Israel; ^3^ Faculty of Health Sciences, Ben Gurion University of the Negev, Beer Sheva, Israel; ^4^ Department of Hematopoietic Biology and Malignancy, The University of Texas MD Anderson Cancer Center, Houston, TX, United States; ^5^ Department of Laboratory Medicine, Division of Pathology/Laboratory Medicine, The University of Texas MD Anderson Cancer Center, Houston, TX, United States; ^6^ School of Health Professions, The University of Texas MD Anderson Cancer Center, Houston, TX, United States; ^7^ Department of Hematopathology, The University of Texas MD Anderson Cancer Center, Houston, TX, United States; ^8^ Division of Hematology/Oncology, Department of Medicine, Chao Family Comprehensive Cancer Center, University of California, Irvine, CA, United States; ^9^ Center of Excellence in Applied Epidemiology, Faculty of Medicine, Thammasat University, Pathumthani, Thailand

**Keywords:** signal regulatory protein alpha, mismatch, relapse protection, cGVHD, HSCT, lymphoid malignancies, innate immunity

## Abstract

Allogeneic hematopoietic stem cell transplantation (allo-HSCT) is a potentially curative therapy for hematologic malignancies. Alloreactivity after HSCT is known to be mediated by adaptive immune cells expressing rearranging receptors. Recent studies demonstrated that the innate immune system could likewise sense the non-self signals and subsequently enhance the alloimmune response. We recently demonstrated that the donor/recipient mismatch of signal regulatory protein α (SIRPα), an immunoglobulin receptor exclusively expressed on innate cells, is associated with a higher risk of cGVHD and relapse protection in a cohort of acute myeloid leukemia patients who underwent allo-HSCT. Whether these effects also occur in other hematologic malignancies remains unclear. In the present study, we compared outcomes by SIRPα match status in a cohort of 310 patients who received allo-HSCT from an HLA matched-related donor for the treatment of lymphoid malignancies. Multivariable analysis showed that SIRPα mismatch was associated with a significantly higher rate of cGVHD (hazard ratio [HR] 1.8, P= .002), cGVHD requiring systemic immunosuppressive therapy (HR 1.9, P= .005), a lower rate of disease progression (HR 0.5, P= .003) and improved progression-free survival (HR 0.5, P= .001). Notably, the effects of SIRPα mismatch were observed only in the patients who achieved >95% of donor T-cell chimerism. The mismatch in SIRPα is associated with favorable relapse protection and concurrently increased risk of cGVHD in patients who undergo allo-HSCT for lymphoid malignancies, and the optimal donor could be selected based on the finding of the study to mitigate the risk of GVHD and relapse.

## Introduction

Allogeneic hematopoietic stem cell transplantation (allo-HSCT) is curative for several high-risk hematologic malignancies. Despite the remarkable advances made over the past two decades with notable improvement in overall survival (OS), relapse and graft-versus-host disease (GVHD) remain frequent causes of failure and death after transplant. It has been long known that the success of allo-HSCT is largely based on achieving a balance between graft-versus-tumor (GVT) and graft-versus-host effects. Hence, regulating the alloimmune response to reduce relapse without increasing GVHD remains critical to improving disease control without increasing non-relapse mortality (NRM). Additionally, a considerable rate of GVHD persists in allo-HSCT recipients with human leukocyte antigen (HLA)-matched related donors (1), indicating that alloreactivity derived from non-HLA genetic variation might play a role in regulating alloimmunity in allo-HSCT recipients.

Signal regulatory protein α (SIRPα) is a polymorphic transmembrane protein with three immunoglobulin domains. SIRPα is exclusively expressed on innate immune cells, including monocytes, macrophages, and myeloid cells, whereas its ligand CD47, is expressed ubiquitously. The interaction of SIRPα and CD47 elicits an inhibitory signal and suppresses macrophage phagocytic function (2). A study using a murine model with marrow transplantation showed that a mismatched SIRPα between donor and recipient was associated with increased allorecognition response followed by enhanced monocyte activation and dendritic cell transformation. The alloreactive response was likely elicited by the non-self signaling that occurs when the SIRPα variant introduced with the allograft binds to CD47 with a different affinity (3). Additionally, Jardine et al. showed that human GVHD lesions are predominately infiltrated with donor monocyte-derived macrophages, which enhanced the proliferation and activation of allogeneic T cells. Although its role in GVHD pathogenesis needs to be further clarified, SIRPα was shown to be significantly upregulated in GVHD macrophages (4). Several specific variations in human SIRPα have been identified, the prevalence and clinical effects of donor/recipient SIRPα variant mismatch on HSCT, as well as the underlying cellular mechanisms of these effects, need to be further investigated.

In a recent study of patients with acute myeloid leukemia (AML) or myelodysplastic syndrome (MDS) who underwent HSCT from HLA-matched related donors, we found that SIRPα variant mismatch between donor and recipient pairs was relatively common, and SIRPα mismatch was associated with a significantly higher risk of chronic GVHD (cGVHD) and lower risk of relapse (5). SIRPα variant mismatch was associated not only with a higher rate of cGVHD and *de novo* cGVHD but also increased severity of cGVHD, indicated by the incidence of cGVHD requiring therapy (5). It is plausible that SIRPα mismatch in allo-HSCT elicits non-self recognition and monocyte activation due to the different SIRPα-CD47 binding between the donor and the recipient. This enhanced innate immunity could further promote adaptive immunity and subsequently lead to a higher risk of cGVHD, accompanied by a lower risk of relapse. It is also unclear whether the observed clinical effects of SIRPα variant mismatch extend to populations beyond those receiving allo-HSCT for AML/MDS. In the present study, we examined the effects of SIRPα variant mismatch on clinical outcomes in patients receiving allo-HSCT from HLA-matched related donors for lymphoid malignancies.

## Methods

### Patient Population

This retrospective analysis included adult patients who underwent allo-HSCT for the treatment of lymphoid malignancies at The University of Texas MD Anderson Cancer Center (UTMDACC) between January 2008 and December 2018. Lymphoid malignancies included acute lymphoblastic leukemia (ALL), chronic lymphoblastic leukemia (CLL), Hodgkin’s disease (HD), and non-Hodgkin’s lymphoma (NHL). All patients included received peripheral blood stem cells from an HLA-matched adult sibling donor, and had donor and patient DNA samples available for SIRPα testing. We excluded patients who failed to engraft as well as those who received post-HSCT cyclophosphamide as GVHD prophylaxis, which has a profound effect on GVHD outcomes compared with conventional GVHD-prevention regimens. All patients provided written informed consent for HSCT according to the Declaration of Helsinki. The study protocol was approved by the Institutional Review Board of the UTMDACC.

### SIRPα Variant Typing and Identification of Mismatch

SIRPα typing was performed as previously described (5). Briefly, we used three sets of SIRPα-specific targeting primers, and each 20-µL polymerase chain reaction included 2 µL of tested DNA (20 ng/µL), 4 µL of primer mix, 13.9 µL of LABType Primer Set Dmix (LTPDMX-B; One Lambda, Canoga Park, CA), and 0.1 µL of Tag polymerase. The polymerase chain reaction was conducted at 96°C for 2 minutes, at 10× (96°C for 10 seconds, 63°C for 1 minute) and 20× (96°C for 10 seconds, 59°C for 50 seconds, 72°C for 30 seconds). A total of 20 µL of the product was run on a 2% agarose gel by electrophoresis, along with controls. Typing was determined by the presence or absence of specific amplicons along with positive and negative controls. Similar to the previous study (5), SIRPα variants were identified and separated into two categories with different CD47 binding interfaces. The SIRPα VI category included SIRPα v1, v4, v5, v6, and v9, and the SIRPα VII category included SIRPα v2, v3, v7, v8, and v10 (5–7). The proportion of donors and recipients in each genotype category (VI/VI, VI/VII, VII/VII) are summarized in **Supplementary Table 1**. The matching or mismatching status on either single alleles or both alleles was determined by the donor and recipient typing results. The direction of mismatch was classified by the presence of “non-self” SIRP VII in the host or donor genotype. Therefore, the donor mismatch group included three types of mismatch (donor versus host): VI/VII → VI/VI, VII/VII → VI/VI, and VII/VII → VI/VII, and the host mismatch group included three other types of mismatch (donor versus host): VI/VI → VII/VII, VI/VII → VII/VII, and VI/VI → VI/VII.

### Clinical Endpoints

The primary outcomes were the incidence of grade 2-4 acute GVHD (aGVHD), cGVHD, and cGVHD requiring systemic immunosuppressive therapy (T-cGVHD). Secondary outcomes were grade 3-4 aGVHD, OS, incidence of disease progression, NRM, and progression-free survival (PFS). Time to neutrophil engraftment was also compared according to SIRPα mismatch. If GVHD with strictly aGVHD features was observed after day 100 after HSCT, this was considered late aGVHD, not cGVHD. OS was defined as the time from HSCT to death from any cause. PFS was defined as the time from HSCT to disease progression or death from any cause. NRM was defined as death without evidence of persistence or progression of malignancy. Surviving patients were censored at the time of the last follow-up. Disease progression was defined as evidence of recurrence or progression of malignancy. Time to neutrophil engraftment was defined as the first of 3 consecutive days with an absolute neutrophil count >500/µL. Myeloablative and nonmyeloablative HSCT conditioning regimens were defined according to the Center for International Blood and Marrow Transplant Research operational guidelines (8). T cell chimerism testing was performed as previously described (9).

### Statistical Methods

Patient-, disease-, and HSCT-related baseline factors were compared using the chi-square or Fisher’s exact test, as appropriate, for categorical variables; the Wilcoxon rank-sum test was used to compare continuous variables. The main effect evaluated in association with outcomes was donor/recipient SIRPα variant match or mismatch status. The cumulative incidences of GVHD, disease progression, and NRM was estimated accounting for competing risks which included death or disease relapse for GVHD, death of any cause before progression for disease progression, and disease progression or disease-related death for NRM. In addition, a diagnosis of grade 1-4 aGVHD was considered a competing risk for *de novo* cGVHD. PFS and OS were estimated using the Kaplan-Meier method.

Predictors of outcomes were evaluated in univariable and multivariable analyses using competing risk regression for GVHD, disease progression, and NRM, and Cox proportional hazards regression was used to evaluate predictors of PFS and OS. SIRPα variant match or mismatch status was forced in all multivariable models, irrespective of statistical significance in the univariable analysis. All other predictors that were significant in the univariable analysis were included in the multivariable analysis. Backward elimination was used to develop multivariable prognostic models. First-degree interaction effects between SIRPα variant match or mismatch status and predictors that were found to be significant in the univariable analysis were evaluated and accounted for when indicated. The only significant interaction effect we identified was for diagnosis and disease progression and progression-free survival. For these outcomes, the impact of SIRPα mismatch was seen in subgroup analyses for each of the lymphoid malignancies included in the study, except for NHL. We adjusted for this interaction effect in multivariate analysis. Notably, we did not identify significant interaction effects between SIRPα mismatch and diagnosis for survival or GVHD outcomes. The proportional hazards assumption was evaluated and was not found to have been violated. In addition to SIRPα variant matching status, the following factors were evaluated for their association with outcomes: donor-recipient sex, recipient age, HSCT-specific comorbidity index (HCT-CI), diagnosis, disease status at HSCT (chemo-sensitive or chemo-resistant), conditioning regimen (myeloablative or nonmyeloablative), donor-recipient ABO match status and cytomegalovirus (CMV) status. Statistical significance was defined as P < 0.05, and statistical analyses were performed using primarily STATA 14.0 (StataCorp, College Station, TX).

## Results

### Patient Population and Allo-HSCT Characteristics

A total of 310 patients met the inclusion criteria. The median age of recipients was 51 years (range 18-72) and one-fourth (24%) had an HCT-CI score >3 at the time of allo-HSCT. Most patients received allo-HSCT for the treatment of ALL (37%) or NHL (37%), and 83% had the chemo-sensitive disease at the time of transplantation. A non-myeloablative conditioning regimen was used in 48% of patients, and the graft source was peripheral blood from a 10/10 HLA-matched related donor for all patients. All patients underwent GVHD prophylaxis with tacrolimus and methotrexate. The median transplant year was 2011 (range: 2008-18). These characteristics were similar between patients with (n=130) and without (n=180) SIRPα variant mismatch with their donors (**Table 1**).

**Table 1:** Characteristics of the study population overall and according to donor/recipient SIRPα matching.

		Donor/recipient SIRPα		
	Overall n = 310	Matched n = 180	Mismatched n = 130	*P*
**Recipient age, years** Median (range)	51 (18-72)	51 (19-72)	52 (18-70)	*0.8*
**HCT-CI, n (%)** ≤3 >3	235 (76) 75 (24)	138 (77) 42 (23)	97 (75) 33 (25)	*0.7*
**Donor/Recipient gender, n (%)** Male/Male Female/Female Male/Female Female/Male	116 (37) 61 (20) 49 (16) 84 (27)	68 (38) 32 (18) 25 (14) 55 (31)	48 (37) 29 (22) 24 (18) 29 (22)	*0.3*
**Donor/Recipient CMV status, n (%)** NR/NR R/R NR/R R/NR	32 (10) 169 (55) 80 (26) 26 (8)	19 (11) 98 (55) 40 (23) 20 (11)	13 (10) 71 (55) 40 (31) 6 (5)	*0.04*
**Donor/Recipient ABO, n (%)** Matched Minor mismatch Major mismatch Bidirectional	208 (67) 44 (14) 16 (5) 41 (13)	125 (69) 24 (13) 10 (6) 21 (12)	83 (64) 20 (15) 6 (5) 20 (15)	*0.7*
**Diagnosis, n (%)** Acute lymphoblastic leukemia Chronic lymphoblastic leukemia Non-Hodgkin’s lymphoma Hodgkin’s lymphoma	115 (37) 59 (19) 114 (37) 22 (7)	63 (35) 33 (18) 72 (40) 12 (7)	52 (40) 26 (20) 42 (32) 10 (8)	*0.6*
**Response prior to transplant, n (%)** Chemo-sensitive Chemo-refractory	259 (83) 51 (16)	146 (81) 34 (19)	113 (87) 17 (13)	*0.2*
**Conditioning intensity, (%)** Non-myeloablative Not non-myeloablative	150 (48) 160 (52)	86 (48) 94 (52)	64 (49) 66 (51)	*0.8*
**% Donor T cell chimerism at day +30** ≤95 >95 **% Donor T cell chimerism at day +100** ≤95 >95	86 (33) 176 (67) 43 (22) 148 (77)	56 (37) 94 (63) 28 (27) 77 (73)	30 (27) 82 (73) 15 (17) 71 (83)	*0.07* *0.1*
**Year of transplant** Median (range)	2011 (2008-18)	2011 (2008-18)	2012 (2008-18)	*0.4*
**Follow-up in surviving patients, months** Median (range)	74 (3-124)	73 (3-124)	74 (4-124)	*N/A*

HCT-CI, HSCT-specific comorbidity index; CMV, cytomegalovirus; NR, non-reactive; R, reactive; allo-HSCT, allogeneic hematopoietic stem cell transplantation; N/A, not applicable. Totals may vary because of missing data.

The median follow-up time in surviving patients was 74 months (range 3-124 months), and most of the events occurred within 3 years after allo-HSCT. At 3-years, the OS rate was 61% (95% confidence interval [CI] 55-66%) and PFS 46% (95 CI 41-52%). The 3-years cumulative incidence of disease progression, NRM, and T-cGVHD was 35% (95 CI 30-41%), 17% (95 CI 13-22%), and 29% (95 CI 24-35%), respectively. At 6 months, the cumulative incidence of grade 2-4 and grade 3-4 aGVHD was 32% (95 CI 27-38%) and 9% (95 CI 7-13%), respectively. Outcomes according to SIRPα variant match or mismatch status are summarized in **Supplementary Table 2**. The impact of the SIRPα mismatch direction between the donor and the host did not differ significantly for any of the outcomes, a mismatch in either direction was therefore counted as a mismatch in our analysis. Time to neutrophil engraftment was not associated with SIRPα match or mismatch status (hazard ratio [HR] 1.1, P= .6).

### aGVHD

**Figure 1:**
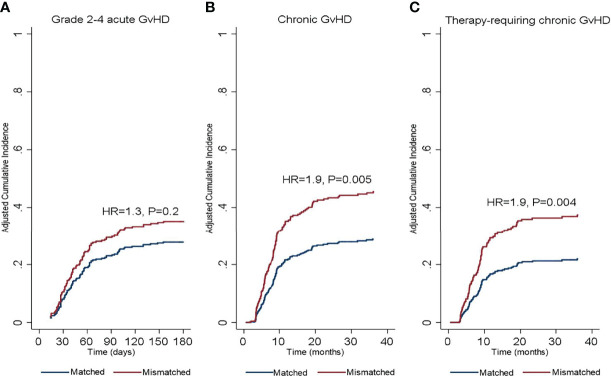
Cumulative incidence of **(A)** grade 2-4 acute graft-versus-host disease, **(B)** chronic graft-versus-host disease, and **(C)** therapy requiring chronic graft-versus-host disease according to donor/recipient SIRPα match or mismatch status. HR, hazard ratio.

In the univariate analysis (**Table 2**), SIRPα mismatch was not associated with the rate of grade 2-4 (HR 1.2, P= .3) or grade 3-4 (HR, 0.5; P= .3) aGVHD at 6-months. Multivariable analysis confirmed the lack of association (HR 1.3, P= .2) between SIRPα variant mismatch and grade 2-4 aGVHD (**Figure 1A**). Female to male HSCT (HR 1.6, P= .03) and the use of a myeloablative conditioning regimen (HR 1.8, P= .004) were the only significant predictors of grade 2-4 aGVHD (**Table 3**). Predictors of grade 3-4 aGVHD were not evaluated in the multivariable analysis given the small number of events.

**Table 2:** Univariable analysis evaluating predictors of grade 2-4 acute GVHD, chronic GVHD and chronic GVHD requiring immunosuppressive therapy.

	N	Grade 2-4 acute GVHD D180		Chronic GVHD 3 yrs		Chronic GVHD requiring IS therapy 3 yrs	
	310						
		HR (95% CI)	*P*	HR (95% CI)	*P*	HR (95% CI)	*P*
**SIRPα**							
Matched	180	1.0		1.0		1.0	
G mismatch	66	1.2 (0.8-2)	*0.4*	1.6 (0.9-2.5)	*0.05*	1.7 (0.9-2.8)	*0.05*
H mismatch	64	1.2 (0.8-2)	*0.4*	1.7(1.1-2.7)	*0.01*	1.7 (1.02-2.9)	*0.04*
G/H Mismatched vs matched	130	1.2 (0.8-1.8)	*0.3*	1.6 (1.1-2.4)	*0.008*	1.7 (1.1-2.6)	*0.01*
**Recipient age, years**							
≤50	143	1.0		1.0		1.0	
>50	167	0.8 (0.6-1.2)	*0.3*	1.02 (0.7-1.5)	*0.9*	0.9 (0.6-1.5)	*0.9*
**HCT-CI, n (%)**							
≤3	235	1.0		1.0		1.0	
>3	75	1.1 (0.7-1.6)	*0.8*	0.8 (0.5-1.3)	*0.4*	0.7 (0.4-1.2)	*0.2*
**Donor / Recipient gender**							
Male / Male	116	1.0		1.0		1.0	
Female / Female	61	0.8 (0.5-1.5)	*0.6*	1.04 (0.6-1.8)	*0.9*	0.96 (0.5-1.8)	*0.9*
Male / Female	49	0.9 (0.5-1.7)	*0.8*	1.2 (0.7-2.2)	*0.5*	0.9 (0.4-1.9)	*0.7*
Female / Male	84	1.5 (0.9-2.4)	*0.08*	1.9 (1.2-2.9)	*0.005*	1.8 (1.1-2.9)	*0.02*
**Donor / Recipient CMV status**							
NR/NR	32	1.0		1.0		1.0	
R/R	169	1.3 (0.6-2.8)	*0.5*	0.7 (0.4-1.3)	*0.3*	0.9 (0.4-1.9)	*0.8*
NR/R	80	1.3 (0.6-3.1)	*0.4*	1.1 (0.6-2.1)	*0.7*	1.3 (0.6-2.9)	*0.4*
R/NR	26	0.9 (0.3-2.6)	*0.8*	1.5 (0.7-2.9)	*0.3*	1.7 (0.7-3.9)	*0.2*
**Donor/Recipient ABO**							
Matched	208	1.0		1.0		1.0	
Minor mismatch	44	1.05(0.6-1.8)	*0.9*	1.3 (0.8-2.3) 0.9	*0.3*	1.4 (0.7-2.5)	*0.3*
Major mismatch	16	0.3 (0.1-1.4)	*0.1*	(0.4-2.1)	*0.8*	1.2 (0.5-3)	*0.6*
Bidirectional	41	1.05 (0.6-1.8)	*0.8*	1.6 (0.9-2.6)	*0.06*	1.6 (0.9-2.8)	*0.09*
**Diagnosis**							
ALL	115	1.8(1.1-2.9)	*0.02*	0.8 (0.5-1.2)	*0.3*	1.1 (0.6-1.8)	*0.8*
CLL	59	2.0 (1.1-3.5)	*0.01*	1.2 (0.8-2)	*0.4*	1.4 (0.8-2.5)	*0.2*
NHL	114	1.0		1.0		1.0	
HD	22	1.2 (0.5-3.1)	*0.5*	1.6 (0.8-3.1)	*0.2*	1.7 (0.8-3.8)	*0.2*
**Response prior to transplant**							
Chemo-sensitive	259	1.0		1.0		1.0	
Chemo-refractory	51	1.3 (0.8-2.2)	*0.3*	0.9 (0.5-1.5)	*0.7*	1.1 (0.7-2)	*0.6*
**Conditioning intensity**							
Non-myeloablative	150	1.0		1.0		1.0	
Myeloablative	160	1.8 (1.2-2.8)	*0.003*	0.7 (0.5-1.0)	*0.06*	0.8 (0.5-1.2)	*0.3*

GVHD, graft-versus-host disease; IS, immunosuppressive; HR, hazard ratio; CI, confidence interval; ALL, Acute lymphoblastic leukemia; CLL, Chronic lymphoblastic leukemia; NHL, Non-Hodgkin’s lymphoma; HD, Hodgkin’s disease; HCT-CI, HSCT-specific comorbidity index; CMV, cytomegalovirus; NR, non-reactive; R, reactive; allo-HSCT, allogeneic hematopoietic stem cell transplantation. Totals may vary because of missing data.

**Table 3:** Multivariable analysis evaluating predictors of grade 2-4 acute GVHD, chronic GVHD and chronic GVHD requiring immunosuppressive therapy.

Predictor	Grade 2-4 acute GVHD D180		Chronic GVHD 3 yrs			Chronic GvHD requiring IS therapy 3 yrs	
	HR(95% CI)	*P*	HR(95% CI)	*P*		HR(95% CI)	*P*
**SIRPα** Mismatched	1.3 (0.9-1.9)	0.2	1.8 (1.2-2.6)	0.002		1.9 (1.2-2.9)	0.005
**Donor/recipient gender** Female / Male	1.6 (1.05-2.4)	0.03	1.9 (1.3-2.9)	0.001		2.03 (1.3-3.2)	0.002
**Conditioning regimen** Myeloablative	1.8 (1.2-2.7)	0.004	0.7 (0.5-0.9)	0.04		0.8 (0.5-1.2)	0.2

GVHD: graft-versus-host disease; IS: immunosuppressive.

### cGVHD

In the univariate analysis (**Table 2**), SIRPα variant mismatch was associated with a significantly higher rate of cGVHD (HR 1.6, P= .008) and T-cGVHD (HR 1.7, P= .01) at 3 years, and these effects persisted in the multivariable analysis (**Figures 1B, C**). Female-to-male HSCT was also independently associated with a higher rate of cGVHD (HR 1.9, P = .001) and T-cGVHD (HR 2.03, P = .002) at 3 years. In addition, the use of a myeloablative conditioning regimen (HR 0.7, P = .04) was associated with a lower rate of cGVHD (**Table 3**).

### Disease Progression and NRM

**Figure 2:**
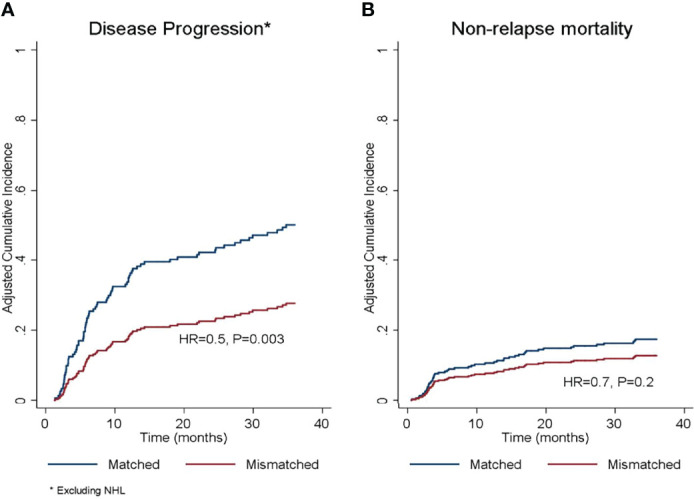
Adjusted cumulative incidence of **(A)** disease progression and **(B)** non-relapse mortality according to donor/recipient SIRPα match or mismatch status. HR, hazard ratio. *The protective effect of SIRPα mismatch was limited to diagnoses other than non-Hodgkin’s lymphoma.

In the univariate analysis (**Table 4**), SIRPα mismatch was protective against disease progression (HR 0.7, P= .05) and this effect persisted in multivariate analysis (HR 0.5, P= .003) (**Table 5** and **Figure 2A**). We further performed subgroup analysis and found that the protective effect of SIRPα mismatch perseveres in each of the lymphoid malignancies included in the study, except for NHL. Our data did not show any effect of SIRPα mismatch on disease progression (HR 1.2, P= .5) for patients with NHL. Stratified analyses showed the median time to relapse in the NHL group was 95 days, which is significantly shorter than that observed for the rest of the cohort (**Supplementary Table 3**). None of the remaining factors evaluated were associated with the rate of disease progression. SIRPα mismatch was not associated with the 3-years NRM rate (**Figure 2B**) in univariable (HR 0.7, P= .3) or multivariable analysis (HR 0.7, P=. 2). Recipient age >50 years (HR 1.8, P= .04) and HCT-CI>3 (HR 2.2, P= .007) were the only significant predictors of NRM in the multivariate analysis (**Table 5**).

**Table 4:** Univariable analysis evaluating predictors of non-relapse mortality, disease progression, progression-free survival and overall survival.

	N	Non-relapse mortality 3 yrs		Disease progression 3 yrs		Disease-free survival 3 yrs		Overall survival 3 yrs	
	310								
		HR (95% CI)	*P*	HR (95% CI)	*P*	HR (95% CI)	*P*	HR (95% CI)	*P*
**SIRPα**									
Matched	180	1.0		1.0		1.0		1.0	
G mismatch	66	0.6 (0.3-1.4)	*0.2*	0.6 (0.4-1.1)	*0.08*	0.6 (0.4-0.9)	*0.04*	0.8 (0.5-1.3)	*0.3*
H mismatch	64	0.8 (0.4-1.7)	*0.6*	0.7 (0.4-1.2)	*0.2*	0.7 (0.5-1.1)	*0.1*	0.8 (0.5-1.3)	*0.4*
G/H Mismatch	130	0.7 (0.4-1.3)	*0.3*	0.7 (0.4-1.0)	*0.05*	0.7 (0.5-0.9)	*0.02*	0.9 (0.5-1.7)	*0.9*
**Recipient age, years**									
≤50	143	1.0		1.0		1.0		1.0	
>50	167	1.9 (1.05-3.4)	*0.03*	0.7 (0.5-1.01)	*0.06*	1.02 (0.7-1.4)	*0.9*	1.1 (0.7-1.5)	*0.7*
**HCT-CI, n (%)**									
≤3	235	1.0		1.0		1.0		1.0	
>3	75	2.2 (1.3-3.9)	*0.01*	1.1 (0.7-1.7)	*0.6*	1.6 (1.1-2.2)	*0.008*	1.8 (1.2-2.7)	*0.002*
**Donor/Recipient gender**									
Male/Male	116	1.0		1.0		1.0		1.0	
Female/Female	61	0.8 (0.4-1.7)	*0.6*	0.8 (0.5-1.4)	*0.4*	1.1 (0.7-1.7)	*0.5*	1.1 (0.7-1.9)	*0.6*
Male/Female	49	1.0 (0.4-2.6)	*0.9*	0.6 (0.3-1.2)	*0.1*	0.8 (0.5-1.4)	*0.5*	0.8 (0.4-1.5)	*0.5*
Female/Male	84	1.4 (0.6-2.9)	*0.4*	0.8 (0.5-1.3)	*0.4*	1.1 (0.7-1.7)	*0.7*	1.2 (0.7-2)	*0.5*
**Donor / Recipient CMV**									
NR/NR	32	1.0		1.0		1.0		1.0	
R/R	169	1.3 (0.5-3.8)	*0.6*	1.4 (0.8-2.7)	*0.3*	1.5 (0.9-2.7)	*0.1*	2.3 (1.1-5)	*0.03*
NR/R	80	1.7 (0.6-5)	*0.3*	0.9 (0.4-1.8)	*0.8*	1.3 (0.7-2.4)	*0.4*	2.0 (0.9-4.5)	*0.09*
R/NR	26	1.2 (0.3-4.9)	*0.7*	0.7 (0.3-2.1)	*0.6*	0.9 (0.4-2)	*0.8*	1.2 (0.4-3.4)	*0.7*
**Donor/Recipient ABO**									
Matched	208	1.0		1.0		1.0		1.0	
Minor mismatch	44	1.5 (0.7-3.1)	*0.2*	0.9 (0.5-1.7)	*0.9*	1.1 (0.7-1.7)	*0.6*	1.3 (0.8-2.1)	*0.4*
Major mismatch	16	0.4 (0.05-2.8)	*0.3*	1.5 (0.7-3.2)	*0.3*	1.03 (0.5-2.1)	*0.9*	1.1 (0.5-2.4)	*0.9*
Bidirectional	41	1.3 (0.6-2.7)	*0.5*	1.3 (0.8-2.3)	*0.3*	1.4 (0.9-2.1)	*0.1*	1.3 (0.8-2.2)	*0.3*
**Diagnosis**									
ALL	115	1.5 (0.8-2.9)	*0.2*	1.6 (0.9-2.5)	*0.06*	1.6 (1.1-2.4)	*0.009*	2.1 (1.4-3.3)	*<0.01*
CLL	59	1.7 (0.8-3.6)	*0.1*	1.1 (0.6-1.9)	*0.7*	1.3 (0.8-2)	*0.2*	1.2 (0.7-2)	*0.5*
NHL	114	1.0		1.0		1.0		1.0	
HD	22	0.7 (0.2-3)	*0.6*	1.2 (0.6-2.7)	*0.6*	1.01(0.5-2)	*0.9*	0.8 (0.3-2)	*0.7*
**Response prior**									
Chemo-sensitive	259	1.0		1.0		1.0		1.0	
Chemo-refractory	51	1.1 (0.5-2.2)	*0.8*	1.5 (0.9-2.4)	*0.06*	1.5 (1.0-2.1)	*0.05*	1.2 (0.8-1.9)	*0.4*
**Conditioning**									
Non-myeloablative	150	1.0		1.0		1.0		1.0	
Myeloablative	160	1.4 (0.8-2.5)	*0.2*	1.4 (1.02-2.2)	*0.04*	1.6 (1.2-2.2)	*0.002*	2 (1.4-2.9)	*<0.01*

GVHD, graft-versus-host disease; HR, hazard ratio; CI, confidence interval; ALL, Acute lymphoblastic leukemia; CLL, Chronic lymphoblastic leukemia; NHL, Non-Hodgkin’s lymphoma; HD, Hodgkin’s disease; HCT-CI, HSCT-specific comorbidity index; CMV, cytomegalovirus; NR, non-reactive; R, reactive; allo-HSCT, allogeneic hematopoietic stem cell transplantation.

**Table 5:** Multivariate analysis evaluating predictors of non-relapse mortality, disease progression, progression-free survival and overall survival.

Predictor	Non-relapse mortality		Disease progression		Disease-free survival		Overall survival	
	HR	P	HR	P	HR	P	HR	P
	(95% CI)		(95% CI)		(95% CI)		(95% CI)	
**SIRPα**
Mismatched	0.7	*0.2*	0.5*	*0.003*	0.5*	*0.001*	0.7	*0.1*
	(0.4-1.3)		(0.3-0.8)		(0.3-0.8)		(0.5-1.1)	
**HCT-CI**
>3	2.2	*0.007*	----	*---*	1.7	*0.003*	1.9	*<0.001*
	(1.2-3.8)				(1.2-2.3)		(1.3-2.9)	
**Age, years**
>50	1.8	*0.04*	----	*---*	----	*---*	----	*---*
	(1.02-3.3)							
**Conditioning regimen**
Myeloablative	----	*---*	----	*---*	1.6	*0.007*	----	*---*
					(1.1-2.2)			
**Diagnosis**
ALL	----	*---*	----	*---*	----	*---*	2.2	*<0.001*
							(1.5-3.2)	

ALL: acute lymphoblastic leukemia* The protective effect of SIRPα mismatch was limited to diagnoses other than non-Hodgkin’s lymphoma. SIRPα mismatch did not impact the rate of disease progression (HR=1.2, 95% CI 0.6-2.5, P= .5) or progression-free survival (HR=1.1, 95% CI 0.6-1.9, P= .8) in patients with non-Hodgkin’s lymphoma.

### PFS and OS

**Figure 3:**
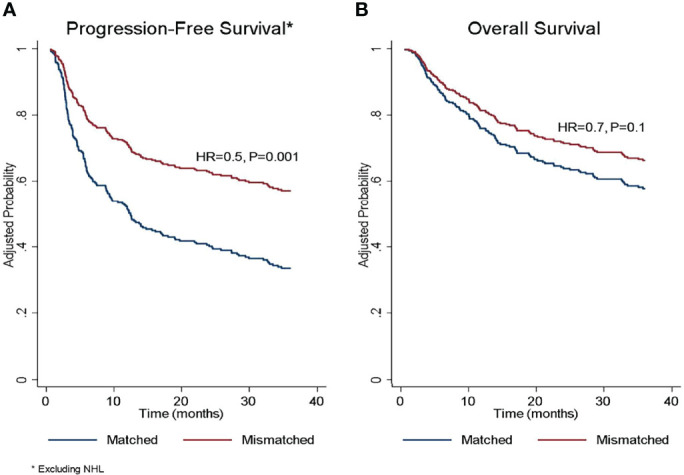
Adjusted probability of **(A)** progression-free survival and **(B)** overall survival according to donor/recipient SIRPα match or mismatch status. HR, hazard ratio; NHL, non-Hodgkin lymphoma. *The protective effect of SIRPα mismatch was limited to diagnoses other than non-Hodgkin’s lymphoma. *The protective effect of SIRPα mismatch was limited to diagnoses other than non-Hodgkin’s lymphoma.

In the univariate analysis (**Table 4**), SIRPα variant mismatch was associated with more favorable PFS (HR 0.7, P= .02), and this effect persisted (HR 0.5, P= .001) in multivariable analysis (**Table 5** and **Figure 3A**). Myeloablative conditioning (HR 1.6, P= .007) and HCT-CI>3 (HR 1.7, P= .003) was associated with worse PFS in the multivariable analysis. For OS, there was no significant impact for SIRPα mismatch (**Figure 3B**) in univariate (HR 0.9, P= .9) but a trend of superior survival in multivariate analysis (HR 0.7, P= .1). HCT-CI>3 (HR 1.9, P<.001) and an ALL diagnosis (HR 2.2, P<.001) were the only two significant predictors of adverse OS in the multivariable analysis (**Table 5**).

### Effects of Donor T Cell Chimerism on SIRPα Variant Mismatch

We performed landmark analyses to evaluate the impact of SIRPα mismatch on disease progression and cGVHD depending on donor T cell chimerism. On day 30 (range 21-45) and day 100 (range 70-145) after allo-HSCT, the impact of SIRPα mismatch was limited to the subset of patients who had >95% donor T cell chimerism (**Supplementary Table 4** and **Supplementary Figures 1A, B**). For patients w >95% donor T cell chimerism at day +30 after HSCT, SIRPα mismatch was associated with a significantly lower rate of subsequent disease progression (HR 0.5, P= .01) and a higher rate of cGVHD (HR 2.1, P= .005). In contrast, for patients with ≤95% donor T cell chimerism at day +30 after HSCT, SIPRα variant mismatch was not associated with disease progression (HR 0.8, P= .5) or cGVHD (HR 0.9, P= .8). Consistent results were observed in the landmark analysis starting at day +100.

## Discussion

There remains an unmet need to improve the cure rates for patients with lymphoid malignancies who failed multiple lines of treatment. Except for a subgroup of patients with B-cell lymphoid malignancies who may benefit from chimeric antigen receptor T-cell therapies, allo-HSCT remains the only potentially curative intervention for these high-risk patients. In this large cohort of patients with lymphoid malignancies who underwent allo-HSCT from MRD, we found that SIRPα variant match or mismatch status is predictive of relapse and GVHD after transplant. In line with the previous study in AML/MDS cohort (5), the results of the study suggested that SIRPα variant mismatch in allo-HSCT could elicit non-self recognition and innate immune activation, which further promotes adaptive immunity and subsequently leads to a reduced risk of relapse and a higher risk of cGVHD. Given the high prevalence and significant clinical impact of donor/recipient SIRPα mismatch, the findings of the present study could have practical applications in best donor selection based on disease risk status. For instance, for patients at high risk of relapse, a mismatched SIRPα donor might be preferred despite the increased risk of cGVHD given the overall improvement in PFS. Instead, for patients who are at low risk for relapse, a matched SIRPα is more reasonable to decrease the risk of cGHVD.

In addition to tumor control function, a growing body of evidence has shown that innate immune activation is crucial for the initiation and persistence of cGVHD, and innate responses were upregulated in patients with cGVHD (10–12). In the present study, the SIRPα mismatch was associated with increased incidence and severity of cGVHD, as evidenced by the increased number of patients requiring systematic therapy, but no impact on aGVHD. It is plausible that the allo-response elicited by the innate cells likely occurs several weeks to months after transplant which is responsible for this impact on cGVHD but not aGVHD (13). The association between the relapse protection and the incidence and severity of GVHD has been reported in several studies of allo-HSCT, but the inverse relationship between GVT and the coupled GVHD is not always consistent among various hematologic malignancies (14, 15). The impact of the SIRPα mismatch on cGVHD observed in the current lymphoid cohort is similar to that seen in the AML/MDS cohort, However, in that study, the relapse protection effect was borderline statistical significant (P = 0.05), whereas a significant association was observed between the presence of SIRPα variant mismatch and reduced relapse (HR 0.5, P = .003) in the present study, which contributed to the significantly improved PFS (HR 0.5, P = .001). These findings indicate that certain lymphoid malignancies may be more sensitive to cGVHD-associated GVT, which could be partially attributable to the innate alloreactivity derived from the SIRPα variant mismatch. In our exploratory subgroup analysis, the trend of increased cGVHD with SIRPα mismatch was seen across all disease subtypes whereas the relapse protection effect was absent in the NHL patients. Of note, the median time to relapse for NHL patients was 95 days after transplant compared to 225 days in all other subgroups (**Supplementary Table 3**), indicating the relapses in NHL may precede the alloreactivity enforced by the recovered innate cells.

Recent data from experimental models have shown that the innate immune system could recognize the non-self signals and prime the immunity against allogeneic grafts (16). Unlike allorecognition mediated by T cells that express rearranging receptors, allorecognition by the innate system appears to be independent of major histocompatibility complex (MHC) mismatch and possibly initiated by the mismatching signal from non-MHC genomic loci (17). In the allo-HSCT setting, it is believed that the host antigens, especially antigens from HLA molecules, are processed and presented to donor T cells by either host or donor Antigen-presenting Cells (APC)s (18), the direction of alloreactivity (GVH or HVG) derived from the HLA mismatch affect the outcomes differently (19). In the present study, all the SIRPα mismatches, regardless of the alloreactive vector or the presence of a specific genotype, are associated with cGVHD and relapse protection. It would be reasonable to postulate that mismatched SIRPα molecules may not be served as an allo-epitope to provoke the adaptive alloimmunity in this scenario, instead, the innate alloresponse activated by the presence of “non-self” SIRPα-CD47 interaction could set up the stage of the subsequential T cell-mediated alloimmunity.

Defining the effector cells and their contributions following the SIRPα mismatch allorecognition would expand our knowledge on the orchestration of innate and adaptive immunity post-HSCT. Consistent with our previous study in AML/MDS, in the present study, the effect of SIRPα mismatch on both cGVHD and relapse protection was observed only in the patients who achieved full (>95%) donor T cell chimerism after allo- HSCT, indicating that enhanced innate immunity may need to activate adaptive immunity first which then leads to a higher risk of cGVHD and a lower risk of relapse. In a mice model that lacked all lymphoid cells, Oberbarnscheidt et al. showed that allograft, but not the syngeneic graft, elicited the differentiation of monocytes into mature DCs, which further stimulated T cell proliferation and IFN-γ production *ex vivo* (16). Studies on the cells influenced by SIRPα-CD47 blockage suggested the tumor control effect is likely attributed to a direct boosting of T cell function and/or an improved APC function [reviewed by Logtenberg et al. (20)]. In the present study, it is possible that donor T cells respond to recipient SIRPα as an alloantigen, and thus create a late-appearing set of T cells enacting both cGVHD and tumor regression. Additionally, other cells expressing SIRPα may also be involved in the alloimmunity mediating cGVHD and relapse protection in our study. Disruption of the SIRPα-CD47 axis significantly enhanced the killing capacity of NK cells, the effect was notably found to be species-specific (21). Moreover, a subset of virus-specific SIRPα^+^ CD8^+^ T cells remained cytolytic function during chronic exhaustion, and programmed cell death ligand (PD-1) blockage expanded this particular subset (22). Future studies are necessary to generate effector donor T cells *in vitro* and assess their specific effector functions for tumor cells and host tissues.

The antibodies targeting CD47 and SIRPα, either alone or in combination with tumor cell-specific opsonizing antibodies and T-cell checkpoint inhibitors, have shown promise in several trials for various malignancies (23–26). A recent study provided substantial evidence suggesting that blockage SIRPα/CD47 axis could enhance adaptive immunity and prime an anti-tumor cytotoxic T-cell response (20). Using an *in vivo* CRISPR screening approach, Manguso et al. showed that loss of CD47 significantly improved tumor control in melanoma cells treated with GVAX and anti-PD-1 immunotherapy in a T-cell-dependent process (27). The persistent alloresponse created from the SIRPα mismatch signal after HSCT could behave similarly to that effect derived from SIRPα/CD47 blockade, and this may work synergistically with other antitumor immunotherapy or immune regulator cell infusions. Further studies are warranted to elucidate the underlying mechanisms and to define the specific role of SIRPα mismatch in an allo-HSCT setting.

The present study was limited by its retrospective nature and the relatively small number of patients with heterogeneous lymphoid malignancy subtypes. The use of the National Institutes of Health criteria was not universally adopted for scoring cGVHD during the study period; hence we assessed the cGVHD severity by the rate of cGVHD requiring systematic treatment. This might negatively impact the reproducibility of our findings in future studies. Additionally, minor H antigens are created by mismatched nonsynonymous single-nucleotide polymorphism (nsSNP), and T cell responses against relevant minor H antigens are generally restricted by certain HLA genotypes (28). Our findings may be influenced by certain nsSNP that impact cGVHD and relapse outcomes. However, most of the studies showing an association between minor H antigen disparity and clinical outcome in allo-HSCT are relatively small, and few of the associations were confirmed in a large multi-institutional study (28, 29).

In conclusion, in our analysis of patients who underwent allo-HSCT from MRD for lymphoid malignancies, we found that SIRPα mismatch was commonly detected in donors/recipient pairs and was significantly associated with a lower rate of relapse, improved PFS, and increased risk of cGVHD. Future prospective studies are needed to validate our findings and to investigate the impact of SIRPα in allo-HSCT with other donor sources. The results of the present study, not only provide valuable information regarding donor choice but also advance our knowledge of allorecognition of the innate immune system in the context of allo-HSCT.

## Data Availability Statement

The raw data supporting the conclusions of this article will be made available by the authors, without undue reservation.

## Ethics Statement

The studies involving human participants were reviewed and approved by The Institutional Review Board of The University of Texas MD Anderson Cancer Center. The ethics committee waived the requirement of written informed consent for participation.

## Author Contributions

RS, SS, RC, and JZ designed the study and contributed to data collection and interpretation and manuscript writing. RS, SSr, UG, and JZ wrote the initial draft of the manuscript. RS designed and performed the statistical analysis, interpreted results, and reviewed and approved the manuscript. SSr, UG, QM, YC, MM, JW, PK, DL, SSa, and KC contributed to data collection and data analysis and reviewed and approved the manuscript. MM, and JW performed the SIRPα variation typing and mismatch interpretation. GR contributed to data collection and reviewed and approved the manuscript. SC, PK, and KC contributed to data interpretation and reviewed and approved the manuscript. SSr, UG, AA, KR, ES, and RC contributed to the treatment of patients and reviewed, edited, and approved the final version of the manuscript. All authors contributed to the article and approved the submitted version.

## Conflict of Interest

The authors declare that the research was conducted in the absence of any commercial or financial relationships that could be construed as a potential conflict of interest.

## Publisher’s Note

All claims expressed in this article are solely those of the authors and do not necessarily represent those of their affiliated organizations, or those of the publisher, the editors and the reviewers. Any product that may be evaluated in this article, or claim that may be made by its manufacturer, is not guaranteed or endorsed by the publisher.
