# Supplementary material for: SIRPα Mismatch Is Associated With Relapse Protection and Chronic Graft-Versus-Host Disease After Related Hematopoietic Stem Cell Transplantation for Lymphoid Malignancies
Source: Front Immunol. 2022 Jul 7;13:904718. doi: 10.3389/fimmu.2022.904718 (PMC9301275; doi:10.3389/fimmu.2022.904718)

Supplemental Table 1. Summary of the proportion of donors and recipients in each SIRPα genotype category

Supplemental Table 2. Univariate estimates of outcomes of allo-HCT according to donor/recipient SIRPα variant match or mismatch status

Supplemental Table 3. Time between allo-HCT and diagnosis of disease progression and chronic GVHD stratified by specific lymphoid diagnosis.

Supplemental Table 4. Time landmark analyses of disease progression and cGVHD depending on donor T cell chimerism

Supplemental Figure 1. Cumulative incidence of relapse according to SIRPα matching status and percentage of donor chimerism at 30 days after hematopoietic stem cell transplantation. (A) Donor T cell chimerism >95% (B) Donor T cell chimerism ≤95%.

Supplemental Table 1. Summary of the proportion of donors and recipients in each SIRPα genotype category

Genotype	Patient (N=310)	Donor (N=310)
VIVI	122 (39.4%)	124 (40%)
VIVII	147 (47.4%)	142 (45.8%)
VIIVII	41 (13.2%)	44 (14.2%)

Supplemental Table 2. Univariate estimates of outcomes of allo-HCT according to donor/recipient SIRPα variant match or mismatch status

Outcomes of allo-HCT	SIRPα		
	Matched<br>% (95% CI)	Mismatched<br>% (95% CI)	*P*
Incidence of grade 2-4 acute GVHD, 6 months	29% (23-37)	35% (28-45)	*0.3*
Incidence of grade 3-4 acute GVHD, 6 months	8% (5-15)	10% (6-15)	*0.3*
Incidence of chronic GVHD, 3 yrs	31% (24-38)	45% (37-55)	*0.006*
Incidence of therapy requiring chronic GVHD, 3 yrs	23% (18-31)	37% (29-46)	*0.001*
Incidence of non-relapse mortality, at 3 yrs	19% (14-25)	14% (9-22)	*0.3*
Incidence of disease progression, at 3 yrs	41% (34-49)	28% (21-37)	*0.05*
Probability of overall survival, 3 yrs	58% (50-65)	64% (55-72)	*0.9*
Probability of progression-free survival, 3 yrs	39% (32-47)	56% (47-64)	*0.02*

Abbreviations: allo-HSCT, allogeneic hematopoietic stem cell transplantation; GVHD, graft-versus-host disease; CI, confidence interval.

Supplemental Table 3. Time between allo-HCT and diagnosis of disease progression and chronic GVHD stratified by specific lymphoid diagnosis.

Diagnosis	No of Patient			Time to Progression (days)*			Time to cGVHD (days)*		
	Overall	Relapse	cGVHD	25th	50th	75th	25th	50th	75th
NHL	114	34	48	56	**95**	197	169	269	578
ALL	115	51	35	99	175	357	166	253	282
CLL	59	24	26	225	417	995	166	260	473
HD	22	9	11	191	363	382	147	210	280
ALL/CLL/HD combined	196	84	72	130	225	622	163	250	330

Abbreviations: cGVHD, chronic graft-versus-host disease; NHL, non-Hodgkin lymphoma; ALL, acute lymphoblastic leukemia; CLL, chronic lymphoblastic leukemia; HD, Hodgkin disease.
*Twenty-fifth, fiftieth, and seventy-fifth percentiles are shown.

Supplemental Table 4. Time landmark analyses of disease progression and cGVHD depending on donor T cell chimerism

Time landmark	Donor T cell chimerism	Progression			cGVHD		
		No.	HR (95% CI) SIRPα MM vs M	P	N	HR (95% CI) SIRPα MM vs M	P
Day 30 after allo-HSCT	≤95%	86	0.8 (0.4-1.7)	0.5	86	0.9 (0.4-1.8)	0.8
	>95%	176	0.5 (0.3-0.9)	0.01	176	2.1 (1.2-3.5)	0.005
Day 100 after allo-HSCT	≤95%	43	0.7 (0.2-2.1)	0.5	42	0.7 (0.2-1.9)	0.4
	>95%	148	0.4 (0.2-0.8)	0.01	146	2.3 (1.4-3.9)	0.001

Abbreviations: cGVHD, chronic graft-versus-host disease; HR, hazard ratio; CI, confidence interval.

Supplemental Figure 1.

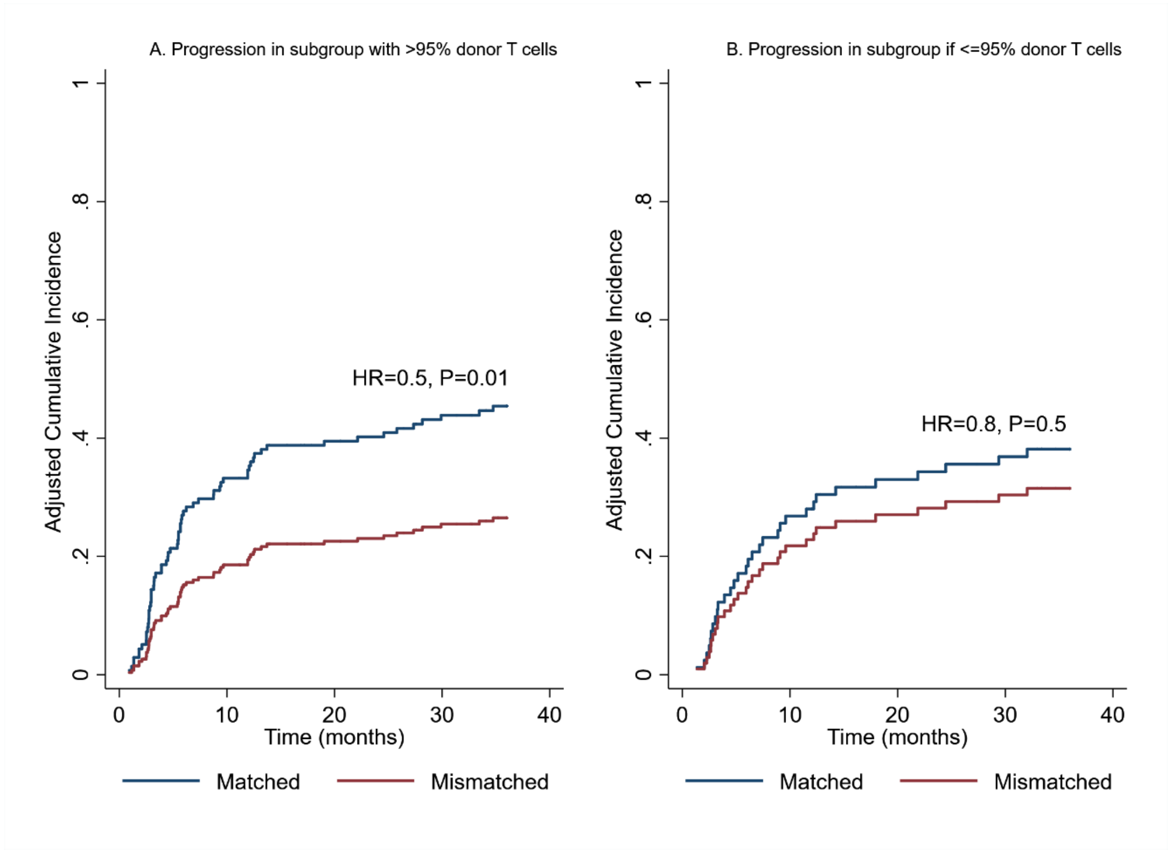

Supplement: Supplementary file 1 [file DataSheet_1.pdf]
